# Src and STAT3 inhibitors synergize to promote tumor inhibition in renal cell carcinoma

**DOI:** 10.18632/oncotarget.5971

**Published:** 2015-11-26

**Authors:** Hui-Wen Lue, Brook Cole, Soumya A. M. Rao, Jennifer Podolak, Ahna Van Gaest, Carly King, Christopher A. Eide, Beth Wilmot, Changhui Xue, Paul T. Spellman, Laura M. Heiser, Jeffrey W. Tyner, George V. Thomas

**Affiliations:** ^1^ OHSU Knight Cancer Institute, Oregon Health and Science University, Portland, OR 97239, USA; ^2^ Biomedical Engineering, Oregon Health and Science University, Portland, OR 97239, USA; ^3^ Hematology and Oncology, Oregon Health and Science University, Portland, OR 97239, USA; ^4^ Howard Hughes Medical Institute, Oregon Health and Science University, Portland, OR 97239, USA; ^5^ Oregon Clinical and Translational Research Institute, Oregon Health and Science University, Portland, OR 97239, USA; ^6^ Molecular and Medical Genetics, Oregon Health and Science University, Portland, OR 97239, USA; ^7^ Cell, Developmental and Cancer Biology, Oregon Health and Science University, Portland, OR 97239, USA; ^8^ Pathology and Laboratory Medicine, Oregon Health and Science University, Portland, OR 97239, USA

**Keywords:** cancer, kinase inhibitors, kidney, Src, STAT3

## Abstract

The intracytoplasmic tyrosine kinase Src serves both as a conduit and a regulator for multiple processes required for the proliferation and survival cancer cells. In some cancers, Src engages with receptor tyrosine kinases to mediate downstream signaling and in other cancers, it regulates gene expression. Src therefore represents a viable oncologic target. However, clinical responses to Src inhibitors, such as dasatinib have been disappointing to date. We identified Stat3 signaling as a potential bypass mechanism that enables renal cell carcinoma (RCC) cells to escape dasatinib treatment. Combined Src-Stat3 inhibition using dasatinib and CYT387 (a JAK/STAT inhibitor) synergistically reduced cell proliferation and increased apoptosis in RCC cells. Moreover, dasatinib and CYT387 combine to suppress YAP1, a transcriptional co-activator that promotes cell proliferation, survival and organ size. Importantly, this combination was well tolerated, and caused marked tumor inhibition in RCC xenografts. These results suggest that combination therapy with inhibitors of Stat3 signaling may be a useful therapeutic approach to increase the efficacy of Src inhibitors.

## INTRODUCTION

In cancer cells, Src is activated nonmutationally through its interaction with growth factor receptors and depending on the context, results in proliferation, survival, growth, adhesion, migration, invasion, angiogenesis and metastases [1–6]. Inhibition of Src therefore represents a promising oncologic therapeutic target and several agents, such as dasatinib, bosutinib and saracatinib have been tested in clinical trials. However clinical responses to these agents have been modest, with rare complete and/or durable responses [7–23].

The most commonly used drugs to treat metastatic renal cell carcinoma (RCC) are agents that target VEGF receptors and those that inhibit mTOR. However, while most patients initially respond to these agents the natural history is one of relapse, development of resistance with subsequent disease progression on treatment and death [24–26]. Thus, to address the unmet need to identify additional targets in RCC, our group and others recently identified Src as a novel therapeutic target in RCC [27, 28]. Dasatinib alone, however, was cytostatic and failed to kill RCC cells based on *in vitro* and *in vivo* apoptosis assays [28]. Because of the lack of clinical efficacy of Src inhibitors, our present study sought to identify additional strategies that may increase the effectiveness of Src inhibitors, and importantly reboot the utility of Src inhibitors such as dasatinib in the clinic.

## RESULTS

### Combined inhibition of Src and Stat3 enhances Src pathway inhibition

**Figure 1:**
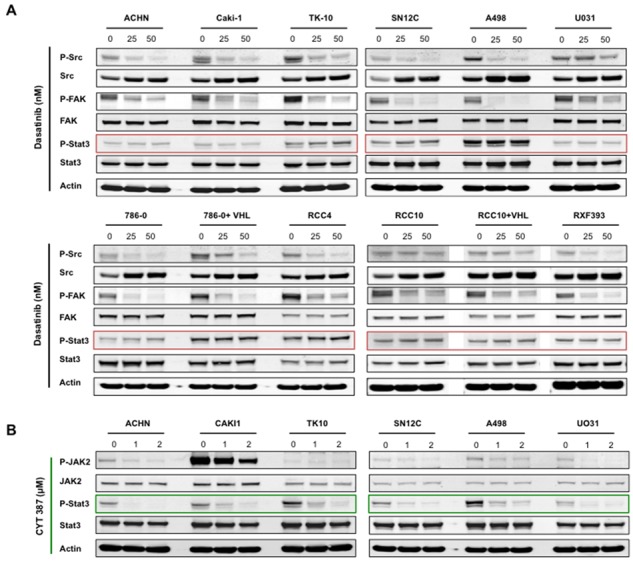
Dasatinib inhibits Src signaling, but not STAT3 activation in RCC cells lines RCC cell lines were treated for 24 hours with the indicated concentrations of either **A.** dasatinib or **B.** CYT387, and lysates were probed with the indicated antibodies. Actin was used as loading control.

**Figure 2:**
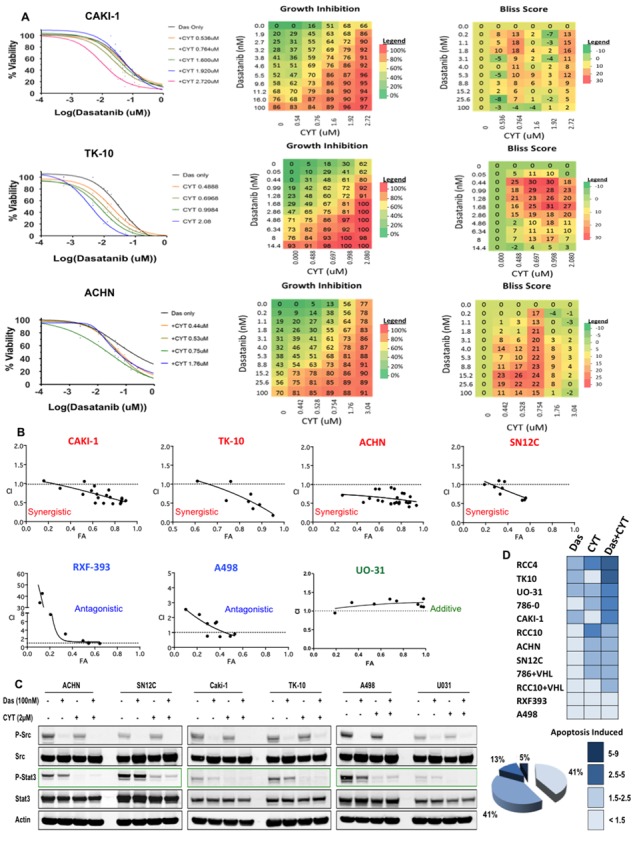
Src and STAT3 are synergistic targets in RCC **A. Left:** Dose response curves in the presence of various doses of CYT387 and dasatinib in Caki-1, TK10 and ACHN RCC cell lines; **Middle:** heatmap of growth inhibition, and **Right:** heatmap of Bliss Scores: CAKI-1: 215; TK10: 621; ACHN: 454. **B.** Growth of RCC cells were analyzed after 5 days of treatment with dasatinib and CYT387. Combination index (CI) were determined by using the Chou-Talalay method (CompuSyn software) for drug combinations with a fractional effect (FA) between 0.2 and 0.9 (20–90% of cell growth inhibition relative to control). CI values < 1 indicates drug synergy. **C.** RCC cells were treated with 100nM of dasatinib and 2 μM of CYT387, alone, in combination or DMSO for 24 hours and lysates were probed with the indicated antibodies. **D.** Twelve RCC cell lines were treated with dasatinib, CYT387 or the combination for 72 hours and apoptotic cells were determined by Caspase 3/7 activation (Caspase-Glo assay). For each cell line, the fold change in apoptosis is color-coded. The percentage of all cell lines exhibiting the indicated degree of apoptosis is shown.

Pre-clinical studies in a wide variety of solid tumors have shown that dasatinib is primarily cytostatic, and this is consistent with the clinical experience, where dasatinib activity is associated with stable disease but complete responses are rarely observed [7–23, 28–33]. Consistent with this, we observed that physiologically relevant doses of dasatinib (~100nM) was effective in reducing the proliferation of the majority of the RCC cell lines (Supplemental Figure 1) [34, 35]. We hypothesized that the purely cytostatic response observed with Src inhibition alone results from bypass survival signaling pathways present in cancer cells that override the therapeutic benefit of dasatinib. Because Stat3 is a known mediator of survival signaling downstream of Src, we decided to test this hypothesis by examining the effect of dasatinib on the levels of phosphorylated Stat3 (hence, activation) [4]. We observed that dasatinib effectively suppressed phosphorylation of Src and its substrate FAK at low concentrations (i.e. 25–100 nM, Figure 1A and Figure 2C). Surprisingly, dasatinib failed to abrogate the phosphorylation of Stat3 in all of the cell lines in our panel, and in some cell lines resulted in higher levels of Stat3 phosphorylation (for example TK10 and SN12C). Stat3 has been shown to promote cell survival and induce drug resistance in cancer cells [34, 36–39]. Together, these findings suggest that although dasatinib effectively dephosphorylates Src, there is persistence of Stat3 signaling, which may mediate dasatinib-independent survival signals.

To test the role of Stat3 in overriding dasatinib inhibition, we treated the RCC cells with CYT387 (Momelotinub ^®^), a JAK-STAT inhibitor that is currently in clinical trials for myeloproliferative neoplasia [40]. Accordingly, CYT387 treatment led to suppression of Stat3 phosphorylation in RCC cells (Figure 1B). We next determined whether the co-targeting of Src and Stat3 exhibited synergistic activity in RCC cancer cells by treating each of the cell lines with increasing concentrations of dasatinib and CYT387 alone and in combination. We used a dose matrix to sample a large range of concentrations and concentration ratios and analyzed combination effects using the Bliss independence model [41]. Positive Bliss scores indicate combination effects where the effect is greater than additive. The heatmaps for CAKI-1, TK-10 and ACHN show that there are positive Bliss scores across a large range of concentration for both compounds (Figure 2A). Next we confirmed our findings using the Chou-Talalay analysis to test for synergy. Dasatinib and CYT387 combinations in 9 out of 12 cell lines (for example, CAKI-1, ACHN, TK10, SN12C) demonstrated a combination index (CI) of less than 1.0, indicating synergy (Figure 2B and Supplemental Figure 2). Stat3 phosphorylation was inhibited in cell lines exhibiting synergy to the dasatinib and CYT387 combinations (for example CAKI-1, TK-10, ACHN and SN12C) as well as those that were additive or antagonistic (for example UO31 and A498, respectively; Figure 2C). These findings suggest that, although inhibition of p-Src and p-Stat3 are clearly necessary, it alone is not sufficient to predict synergy to the combination therapy.

Moreover, combined treatment with dasatinib and CYT387 caused a marked increase in apoptosis in the majority of RCC cells in the panel compared to dasatinib alone (Figure 2D). Together, these data demonstrate that Stat3 inhibition enhanced the effects of dasatinib in RCC cells.

### Combined inhibition of Src and Stat3 reduces tumor growth

**Figure 3:**
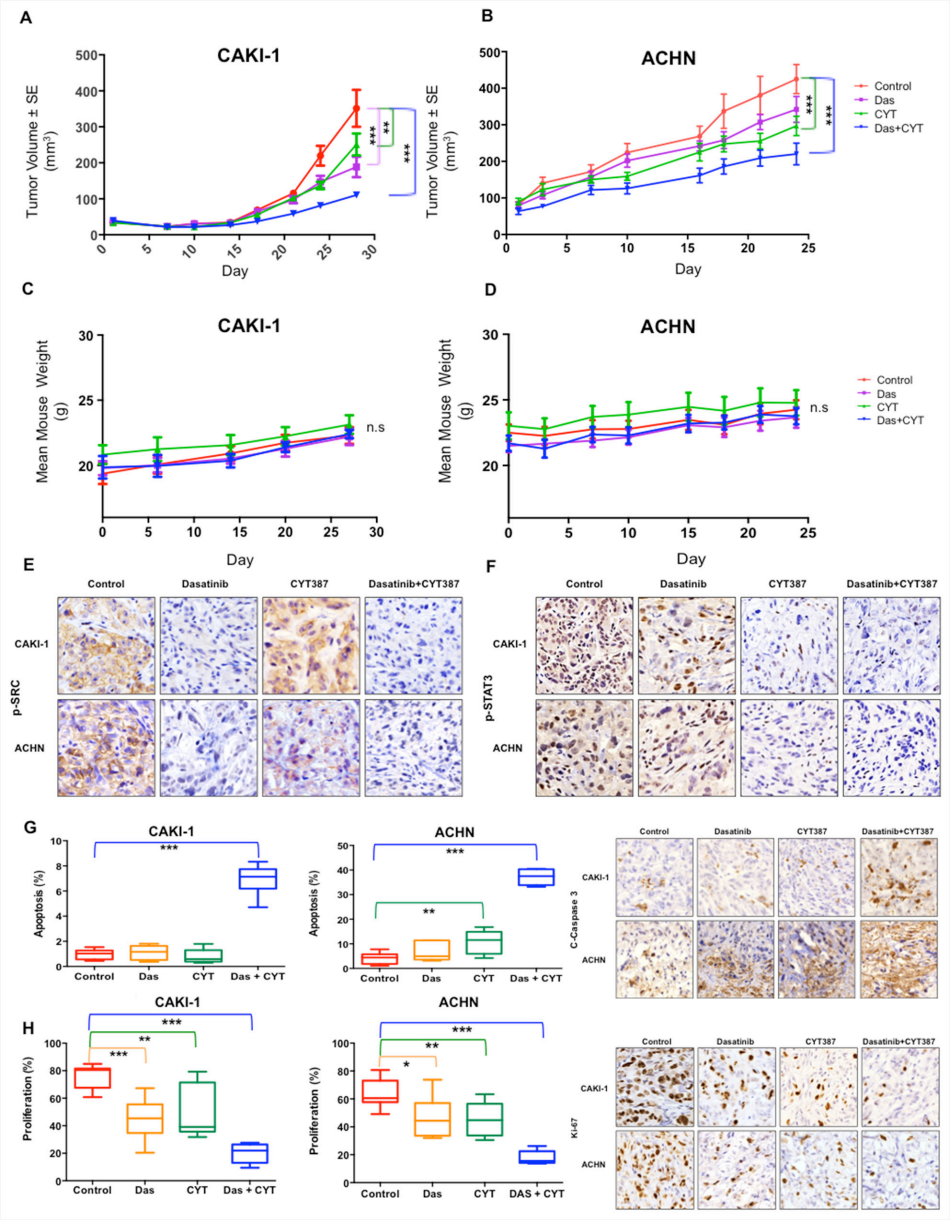
*In Vivo* efficacy of combined Src and STAT3 inhibition **A.** CAKI-1 and **B.** ACHN xenografts were treated with vehicle (control), dasatinib (25mg/kg/day), CYT387 (50mg/kg/day) or both drugs in combination Tumor volume is shown. Error bars represent mean ± SEM. * denotes *P* < 0.05, ** denotes *P* < 0.01, and *** denotes *P* < 0.001. Body weights of mice bearing **C.** Caki-1, and **D.** ACHN tumors as indicated. Data are presented as mean ± SEM. *ns* denotes not significant. **E–H.** Tumor tissue from CAKI-1 and ACHN xenografts treated with the indicated drug regimens were evaluated by immunohistochemistry for (E) p-STAT3; (F) p-SRC; (G) cleaved-Caspase 3 (a marker of apoptosis), and (H) Ki-67 (a marker of proliferation). Error bars represent mean ± SEM. * denotes *P* < 0.05, ** denotes *P* < 0.01, and *** denotes *P* < 0.001.

We next examined the safety and efficacy of inhibiting Src and Stat3 *in vivo* in two xenograft tumor models. While dasatinib or CYT387 alone exhibited anti-tumor effect on CAKI-1 and ACHN xenografts, the combination of dasatinib with CYT387 resulted in significantly greater tumor growth inhibition (71% TGI) in CAKI-1 and (48% TGI) in ACHN tumor xenografts (*p < 0.001;* Figure 3A and 3B, respectively). Importantly, combination treatment was well tolerated, with no weight loss recorded (Figure 3C and 3D, respectively). Pharmacodynamic studies demonstrated that combination therapy led to the suppression of Stat3 and Src phosphorylation (Figure 3E and 3F, respectively). Consistent with our prior finding, dasatinib alone had minimal impact on apoptosis [28]. In marked contrast, combination treatment with dasatinib and CYT387 resulted in significant increase in apoptosis (demonstrated by increase in cleaved-caspase3, *p < 0.001*; Figure 3G) and reduction in proliferation (demonstrated by decrease in Ki-67, *p < 0.001*; Figure 3H). Therefore, prolonged inhibition of Src and Stat3 signaling by combining dasatinib and CYT387 is associated with enhanced inhibition of tumor growth with good tolerability.

### Dasatinib and CYT arrests YAP1 transcriptional programs

**Figure 4:**
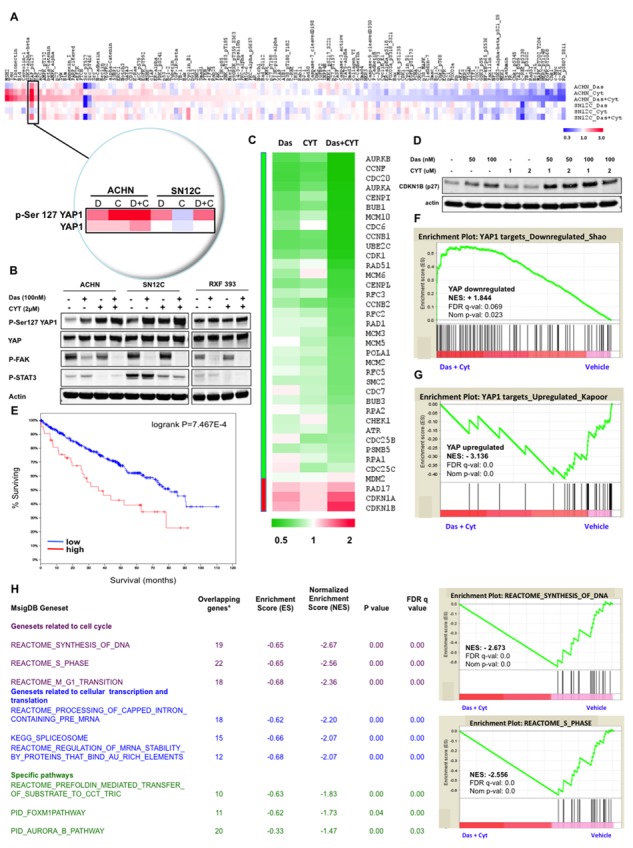
Dasatinib and CYT387 combine to inhibit YAP dependent transcription **A.** Heatmap of total or phosphorylated protein abundance from each of the different samples (ACHN or SN12C that were treated with dasatinib, CYT387 or the combination of both drugs for 24 hrs) determined by RPPA. Insert depicts p-Ser127YAP1 and total YAP1 levels. **B.** Western blots of p-Ser127 YAP1 and total YAP1, p-FAK, p-STAT3 treated with the indicated drugs for 24 hours in ACHN, SN12C and RXF393 RCC cells. **C.** Heatmap of YAP1 regulated genes in ACHN cells after treatment with dasatinib, CYT387 or the combination of both drugs, as analyzed by RNA-Seq. **D.** Western blots of CDKN1B (p27) in ACHN cells. Actin is used as loading control. **E.** Kaplan-Meier survival plots as a function of relative expression of *YAP1* and *TAZ* mRNA in the TCGA kidney cancer cohort of 499 patient tumors (*p* = 7.467e-4). **F–G.** GSEA plots of the ranked list of differentially expressed genes between dasatinib and CYT387 co-treatment (Das+CYT387) and vehicle (Veh), using two genesets: (F) YAP-downregulated, and: (G) YAP-upregulated. **H. Left**: Table of gene sets from MSigDB (C2) collection enriched among genes downregulated by the dasatinib-CYT387 combination treatment in ACHN cells with normalized enrichment score (NES) ranked by FDR *q* value; **Right**: examples of GSEA plots of genes regulating transcription/translation in ACHN cells that are downregulated by the dasatinib and CYT387 combination therapy.

We observed that a lack of response, and subsequently synergy to the dasatinib and CYT387 combination treatment in several cell lines despite robust p-Src and p-Stat3 inhibition that was comparable to that achieved in synergistic cell lines (Figure 2C, 2D). These findings suggest that despite on-target inhibition by dasatinib and CYT387, suppression of Src and Stat3 activation are not sufficient to predict sensitivity to this combination. To identify potential signaling pathways downstream of combined dasatinib+CYT387 treatment that have greater fidelity for predicting synergistic response, we performed reverse phase protein array (RPPA) analysis and the fold changes were translated to a z-score (*see Methods*). Strikingly, we observed that the levels of phosphorylated Serine 127-YAP1 (Yes-associated protein 1) increased by approximately 6- and 2-fold in the combination treatment compared to vehicle control in ACHN and SN12C cells, respectively (Figure 4A).

Phosphorylation at Ser127 of YAP1 results in its sequestration in the cytoplasm and subsequent degradation. YAP1 regulates several context-specific transcriptional programs that promotes tumor proliferation, survival and organ growth, and is an effector of the canonical Hippo signaling pathway that comprises a cascade of kinases that includes the Hippo/MST1–2 kinases, the adaptor Sav1, and the LATS1/2 kinases [42]. When the Hippo signaling is turned on, it culminates in the phosphorylation and consequent inactivation of the transcriptional co-activators YAP1 and transcriptional co-activator with PDZ-binding motif (TAZ) by LATS1/2, which suppresses the TEA domain-containing sequence-specific transcription factors (TEADs)-dependent expression of a network of genes that promote cell proliferation and survival. Studies in mouse models have shown that YAP1 functions as an oncogene [43, 44]. The increase in p-Ser127YAP1 levels in ACHN and SN12C cells, which are both synergistic to the dasatinib-CYT387 combination, was confirmed through Western blot analysis (Figure 4B). Notably, the combination of dasatinib and CYT387 in ACHN and SN12C cells induced greater phosphorylation of Ser127YAP1 than either drug alone, indicating that CYT387 and dasatinib affect YAP1 via a parallel pathway. In contrast, p-Ser127YAP1 levels did not increase in RXF393 cells, which exhibit antagonism to, and are resistant to apoptosis when co-treated with dasatinib and CYT387 (Figure 2B and 2D).

To further investigate the transcriptional responses to YAP1 inactivation, we analyzed the RNA sequencing (RNA-Seq) profiles of ACHN cells treated with dasatinib and CYT387 (Figure 4C). Consistent with the decrease in YAP1 activation, we observed an increase in YAP1 gene targets that encode for inhibition of the cell cycle, such as CDKN1A and CDKN1B; and similarly, a decrease in those that encode for cell cycle progression, such as CDK1, with the combination therapy of dasatinib and CYT387 having greater effect on gene expression than either drug alone [45]. The gain in *CDKN1B (p27)* transcripts in ACHN cells with dasatinib and CYT387 co-treatment corresponded with an increase at the protein level (Figure 4D).

Then in a cohort of 499 patients from the TCGA RCC dataset [46], we found that high levels of *YAP1* and *TAZ* transcripts itself, as well as its regulated genes *CDK1, AURKA, AURKB, CCNB1 and CCNB1* are associated with poor prognosis (Figure 4E and Supplemental Figure 3).

Next, using gene set enrichment analysis (GSEA), we evaluated two canonical transcriptional signatures of YAP1-dependent genes in ACHN cells treated with dasatinib and CYT387 compared to vehicle [47]. Remarkably, the genes downregulated by YAP1 knockdown [48] were significantly enriched by dasatinib and CYT387 combination treatment (Normalized Enrichment Score = +1.84, where positive values indicate correlation; *P* = 0.023; False Discovery Rate = 0.069) (Figure 4F). Correspondingly, the genes that are positively regulated by YAP1 [49] were downregulated and significantly enriched by dasatinib and CYT387 combination treatment (NES = −3.14, where negative values indicate anticorrelation; *P* = 0.0; FDR = 0.0) (Figure 4G), suggesting a strong correlation with YAP1 transcriptional signatures and Src and Stat3 inhibition.

Next, we performed GSEA on the entire Molecular Signature Database (MSigDB: C2 canonical pathway dataset). Genesets related to inhibition of cell cycle, transcription and translation were significantly enriched in the dasatinib and CYT 387 co-treatment suppressed genes (Figure 4H and Supplemental Table 1). Specifically, biological modules associated with YAP1 (e.g. cell cycle checkpoints, synthesis of DNA) were also anticorrelated with dasatinib and CYT387 co-treatment. Together, these data support the observation that Src and Stat3 inhibition confers a selective repression of transcriptional networks induced by YAP1.

## DISCUSSION

The goal of the present study was to identify ways to improve the initial responses to Src inhibitors. Since Src functions primarily as a signaling rheostat, we hypothesized that Src is best targeted in combination with agents that inhibit molecules that signal through, or engage with it. We observed that dasatinib potently inhibited Src signaling, as demonstrated by suppressed Src and FAK phosphorylation but that the activity of Stat3 persisted, probably through input from other pathways. Cumulative evidence supports activation of Stat3 as an oncogenic pathway in many cancers, including RCC [36, 50–53]. Importantly, persistent activation of Stat3 has been implicated in conferring resistance to some anticancer drugs [36, 37, 54, 55]. Stat3 activation potentiates both proliferative and anti-apoptotic signaling and is associated with chemoresistance. In addition, studies in head and neck and non-small cell lung cancer cells have shown reactivation of Stat3 signaling in the presence of dasatinib, indicating that these cancers have become resistant to dasatinib by finding a way to sustain Stat3 signaling [37, 56].

Since developing drugs that target transcription factors such as Stat3 has so far been challenging, small molecule JAK inhibitors represent a plausible clinical alternative to block Stat3 signaling. [36, 39, 55, 57]. We demonstrated that full suppression of Stat3 activation was achieved with the addition of a JAK inhibitor, CYT387. Importantly, CYT387 synergized with dasatinib in cell lines and in mouse xenografts models to inhibit proliferation, induce apoptosis and suppress tumor growth.

An unexpected finding was the pronounced and concordant increase in Ser127YAP1 phosphorylation by dasatinib and CYT38 with the subsequent suppression of multiple YAP1-dependent transcriptional signatures. Previous reports have documented a role for Yes1 (a Src family kinase member) in phosphorylating Y357YAP1, which results in its nuclear localization and transcriptional activation, which was then inhibited by the addition of dasatinib [58]. While the mechanism(s) mediating an increase in the serine-127 phosphorylation of YAP1 and thereby preventing its nuclear translocation is unknown, it may suggest that both dasatinib and CYT387 positively impact the Hippo signaling cascade with resultant inhibition of cell proliferation and growth. These observations are compatible with the known role of YAP1 as transcriptional regulator of cell proliferation and tumor cell survival and importantly, the RNA-Seq analysis of YAP1 target genes in the TCGA RCC dataset suggests that several YAP1-TAZ regulated genes are associated with poor prognosis of RCC patients.

In summary, we observed synergistic activity with dasatinib and CYT387, and these findings point to a strategy to potentially improve the efficacy of Src inhibitors by co-treatment with Stat3 inhibitors.

## MATERIALS AND METHODS

### Cell lines

ACHN, A498, Caki-1, RCC4, RCC10, RXF393, SN12C, TK-10, U031, 786–0 were used in this study and were obtained from the ATCC. RCC10+VHL and 786+VHL were stably transfected with wild type VHL. Cell lines were maintained in Dulbecco's modified Eagle's medium (DMEM) supplemented with 10% fetal bovine serum (FBS) at 37°C in a 5% CO_2_ incubator.

### Cell viability and apoptosis analysis

Cell viability assays were performed by plating 3*10^3^ cells/well in 24-well plates in triplicate and treating the following day with the indicated agent. The experiment was continued for 5 days and then the cells were fixed using 4% formaldehyde and stained for 1 hour with Syto60. Fluorescence was measured and quantified and photographs were obtained using a LiCor Odyssey Infrared Imager. Experimental results are the average of at least three independent experiments.

Apoptosis was determined using Caspase 3/7-Glo assay kit (Promega) following the manufacturer's instructions. Briefly, 1 × 10^3^ cells per well were plated in 96 well plates and cultured for 24 h. Cells were treated with dasatinib, CYT387 and the combination of dasatinib and CYT387 for −72 h, and then 100 μl reagents were added to each well and incubated for 30 min at room temperature. Caspase 3/7 activity was measured using a luminometer. Luminescence values were normalized by cell numbers. The effect of dasatinib, CYT387 and the dasatinib+CYT387 combination on caspase 3/7 activation was assessed as fold of DMSO-treated control cells.

### Drug combination studies

Cells were seeded in 384-well plates at 500 cells/well. After 24 hours, cells were treated with dasatinib (dose range of 0–400 nM) and CYT387 (dose range of 0–3.5 μM) in a 10 by 5 matrix. Cells were treated for 120 hours, and ATP was determined with CellTiter-Glo (Promega). Dasatinib IC_50_ values were calculated from the 6 single-agent doses using a 3-parameter fit with XLFit (IDBS). The Bliss expectation was calculated with the equation (A+B) - (A*B), where A and B are the fractional growth inhibitions induced by agents A and B at a given dose. The difference between Bliss expectation and observed growth inhibition induced by the combination of agents A and B is the “Bliss scores”. “Bliss sum” is the sum of all Bliss scores in a given matrix. Combination indices (CI) were calculated using Calcusyn software. A CI value less than 1.0 is represents a synergistic drug combination.

### Western blotting

Cells were plated in 6 well dishes and treated the following day with the indicated agents. Treatments were for 24 hours, after which cells were washed with ice cold PBS and, unless otherwise indicated, lysed with Additionally, 0.5 mM DTT, 4 μg/mL leupeptin, 4 μg/mL pepstatin, 4 μg/mL aprotinin, RIPA buffer (Sigma). Phosphatase inhibitor cocktail set II and protease inhibitor cocktail set III (EMD Millipore) were added at the time of lysis. Lysates were centrifuged at 15,000g × 10 min at 4 degrees C. Protein concentrations were calculated based on a BCA assay (Thermo Scientific) generated standard curve. Proteins were resolved using the NuPAGE Novex Mini Gel system on 4% to 12% Bis-Tris Gels (Invitrogen). For western blotting, equal amounts of cell lysates (15–20 μg of protein) were resolved with SDS-PAGE, and transferred to membranes. The membrane was probed with primary antibodies, washed, and then incubated with corresponding fluorescent secondary antibodies and washed. The fluorescent signal was captured using LI-COR (Lincoln, NE) Odyssey Imaging System, and fluorescent intensity was quantified using the Odyssey software where indicated. The following antibodies were used for Western blots: p-Src (Y416), Src, p-Fak (Y576/577), Fak, Jak2, p-YAP1 (S127), and YAP1 (Cell Signaling Technologies). p-Jak2 (Y1007/1008), pStat3 (Y705), Stat3 and β-actin (AC15) (Abcam). Ki67 (Dako) and cleaved caspase3 (Cell Signaling Technologies) were used for immunohistochemistry. Dasatinib and CYT387 for *in vitro* and *in vivo* use were purchased from LC Labs and ChemieTek, respectively.

### *In vivo* xenograft studies

4–6 week old mice were utilized for human renal cell carcinoma xenografts. For both ACHN and Caki-1 cell lines 1 × 10^6^ cells were diluted in 50 μl of PBS and 50 μl of Matrigel (BD Biosciences) and were injected subcutaneously into the right and left flank of each mouse.

Tumors were monitored until they reached an average size of 50–80 mm^3^ (approximately 2 weeks), at which point treatments were begun. Dasatinib (25 mg/kg/day) and CYT387 (50 mg/kg/day) were administered by oral gavage 5 day/week. Dasatinib was dissolved in 1-Methyl-2-pyrrolidinone (NMP, sigma)/Propylene glycol (Sigma) and CYT387 was dissolved in NMP/Captisol (Cydex). Tumors and mouse weights were measured twice weekly. At least 6–8 mice per treatment group were included. All mice were euthanized using CO_2_ inhalation followed by cervical dislocation per institutional guidelines at Oregon Health and Science University. Experiments were approved by the Institutional Animal Care and Use Committee at OHSU.

### Immunohistochemistry studies

Immunostaining was performed following deparaffinization and rehydration of the slides, antigen retrieval was performed in a pressure cooker using citrate buffer (pH 6.0) for 4 min. Endogenous peroxidase activity was blocked with 3% hydrogen peroxide for 15 min at room temperature and nonspecific binding was blocked using 10% normal horse serum for 30 min at room temperature. Samples were incubated at room temperature with p-Y416Src rabbit monoclonal antibody (Y416), cleaved caspase 3, p-Y705Stat3 and Ki67. Slides were developed with Vector Immpress rabbit IgG (#MP7401) and Vector Immpress mouse IgG (#MP7400) for 30 min at room temperature. Chromogenic detection was performed using Vector Immpact DAB (#SK4105) for 3 min. Sides were counterstained with hematoxylin. A 3DHistech MIDI Scanner (Perkin Elmer) was used to capture whole slide digital images with a 20x objective. Images were converted to into MRXS files and computer graphic analysis was completed using inForm 1.4.0 Advanced Image Analysis Software (Perkin Elmer).

### Statistical analysis

Mouse tumor size was analyzed by 2-way ANOVA with time and drug as factors, using GraphPad Prism. Mouse weight during treatment was analyzed by repeated measures 2-way ANOVA, with time and drug as factors. A *P* value less than 0.05 was considered statistically significant. Immunohistochemistry: *P*-values were calculated using one-way ANOVA, with Bonferroni's multiple comparison test. * denotes *P* < 0.05, ** denotes *P* < 0.01, and *** denotes *P* < 0.001.

### Reverse phase protein array

Cell lines were either treated with DMSO, 100 nM dasatinib, 2 μM CYT387, or the combination of dasatinib and CYT387 for 24 h. Lysates were prepared as recommended by MD Anderson Cancer Center (Houston, Texas, USA), arrayed on nitrocellulose-coated slides, probed for a standard list of antibodies at the MD Anderson Cancer Center and results were quantified and normalized using their procedure [59]. Genes with insufficient signal were filtered out. From the quantile normalized RPPA data, the fold change values were calculated (the ratio of drug treated above the vehicle treated) for three treatments: dasatinib, CYT387 and combination of dasatinib+CYT387. We calculated the z-score for the protein fold changes from the RPPA data. Z-scores were calculated from the log2 fold change values. Z-score = (log2 fold change of protein—mean of log2 fold changes of all proteins of the array) / standard deviation of log2 fold changes of all proteins of the array. We applied a z-score cutoff of ± 1.96 for the both cell lines. The proteins/phosphoproteins with a z-score of > 1.96 or <–1.96 in both cell lines were selected. The fold change values were visualized by generating the heatmap using MeV software (http://www.tm4.org/mev.html).

### RNA Seq analysis

Total RNA was extracted from ACHN cells treated with 50nM of dasatinib and 2uM of CYT387, alone, in combination or DMSO for 24 hours. Total RNA was extracted with Trizol/CHCl3 (Life Technologies) according to the manufacturer's protocol. The aqueous phase was put through the Qiagen RNEasy kit for cleanup. Sample preparation followed the Agilent SureSelect Strand-Specific mRNA Library Preparation Protocol (Version A.2, September 2013, Agilent Technologies, Santa Clara, CA). Poly-A RNA was purified from 1ug total RNA per sample using two serial rounds of binding to oligo dT magnetic beads. The poly(A) RNA was chemically-fragmented and first-stand cDNA was synthesized using the RNA Seq First Strand Master Mix (Agilent). After purifying the first strand cDNA using AMPure XP beads, second-strand cDNA was synthesized and ends were repaired. A second round of cDNA purification with AMPure XP beads occurred, the 3′ ends were adenylated, followed by adapter ligation and AMPure XP beads purification. Ligated DNA was PCR amplified for 14 cycles and purified again with AMPure XP beads. Quality of the resulting libraries was assessed with an Agilent 2100 Bioanalyzer DNA 1000 Assay. Libraries were sequenced on an Illumina (San Diego, CA) HiSeq as single-end 50bp reads. Sequence data are available at Gene Expression Omnibus with accession number pending.

Fastq files were re-aligned to the human genome GRCh37.70 with the subjunc function in the Subread package allowing for 5 bp indels and all possible junctions [60]. Reads were assigned to genes and counted using the Featurecounts function of subread [61]. The aligned reads are summarized over exons to obtain number of reads mapping to each union exon (count data) using a custom python script. First, a GTF formatted annotation file is parsed to create union exon ranges. Secondly, the exon boundaries and an alignment in SAM format were read and the number of reads falling in each of the defined exonic regions was counted. Samples were investigated for anomalies or differences that would preclude incorporation in to the final dataset using custom scripts in the R statistical programming language (http://www.R-project.org). Very lowly expressed tags i.e. tags which have fewer than 5 counts in total were excluded. Different samples might be sequenced with different depths or depending on the tissue type some genes may be highly expressed. To account for this the count data is normalized using the upper quartile method that adjusts the top 75th percentile across all samples (R package EdgeR, http://www.bioconductor.org).

TCGA KIRC data was stratified by YAP1 and TAZ mRNA expression using the cBio Cancer Genomics Portal platform (http://cbioportal.org).

### Gene set enrichment analysis

Normalized counts/million (cpm) values from RNAseq data were used for calculating the fold change values using the vehicle treated sample as reference. The fold change values for 1.5-fold regulated genes were used for Gene Set Enrichment Analysis (GSEA) using the Pre-ranked option (GSEA-P) in GSEA v2.1.0 software as described in [47]. Analysis was performed on C2-canonical pathways defined in MSigDB (http://www.broadinstitute.org/gsea/msigdb/genesets.jsp?collection=CP) with a weighted enrichment statistic. One thousand permutations of the data were completed to obtain an FDR *q* value. Significance was defined as having an FDR *q* value < 0.25. GSEA was performed independently for three treatments, i.e.: dasatinib, CYT387 and dasatinib+CYT387 and significant genesets were compared.

To specifically look at effect of combination treatment on YAP1, we performed GSEA analysis focusing on YAP1 targets. We defined three genesets as readouts for YAP1 activity:

1. “REACTOME_YAP1_AND_WWTR1_TAZ_STIMULATED_GENE_EXPRESSION” defined as genes involved in YAP1- and WWTR1 (TAZ)-stimulated gene expression from MSigDB,
2. “YAP1_TARGETS_AVNISHKAPOOR” defined as genes upregulated in YAP1 overexpressing mouse pancreatic tumors [49] and, 3) “YAP1_TARGETS_DIANE_SHAO” defined as genes downregulated in HCTtetK cells stably expressing YAP1 (data from GSE55942) as described in [48].

For YAP1, we performed GSEA analysis on all the genes from the RNAseq data using phenotype permutation with a weighted enrichment statistic and a signal-to-noise metric for ranking genes.

## SUPPLEMENTARY FIGURES AND TABLE





## References

[1] Abram CL, Courtneidge SA (2000). Src family tyrosine kinases and growth factor signaling. Exp Cell Res.

[2] Bromann PA, Korkaya H, Courtneidge SA (2004). The interplay between Src family kinases and receptor tyrosine kinases. Oncogene.

[3] Irby RB, Yeatman TJ (2000). Role of Src expression and activation in human cancer. Oncogene.

[4] Kim LC, Song L, Haura EB (2009). Src kinases as therapeutic targets for cancer. Nat Rev Clin Oncol.

[5] Kopetz S, Shah AN, Gallick GE (2007). Src continues aging: current and future clinical directions. Clin Cancer Res.

[6] Yeatman TJ (2004). A renaissance for SRC. Nat Rev Cancer.

[7] Brooks HD, Glisson BS, Bekele BN, Johnson FM, Ginsberg LE, El-Naggar A, Culotta KS, Takebe N, Wright J, Tran HT, Papadimitrakopoulou VA (2011). Phase 2 study of dasatinib in the treatment of head and neck squamous cell carcinoma. Cancer.

[8] Dudek AZ, Pang H, Kratzke RA, Otterson GA, Hodgson L, Vokes EE, Kindler HL, Cancer, Leukemia Group B (2012). Phase II study of dasatinib in patients with previously treated malignant mesothelioma (cancer and leukemia group B 30601): a brief report. J Thorac Oncol.

[9] Finn RS, Bengala C, Ibrahim N, Roche H, Sparano J, Strauss LC, Fairchild J, Sy O, Goldstein LJ (2011). Dasatinib as a single agent in triple-negative breast cancer: results of an open-label phase 2 study. Clin Cancer Res.

[10] Gold KA, Lee JJ, Harun N, Tang X, Price J, Kawedia JD, Tran HT, Erasmus JJ, Blumenschein GR, William WN, Wistuba II, Johnson FM (2014). A phase I/II study combining erlotinib and dasatinib for non-small cell lung cancer. Oncologist.

[11] Gubens MA, Burns M, Perkins SM, Pedro-Salcedo MS, Althouse SK, Loehrer PJ, Wakelee HA (2015). A phase II study of saracatinib (AZD0530), a Src inhibitor, administered orally daily to patients with advanced thymic malignancies. Lung Cancer.

[12] Haura EB, Tanvetyanon T, Chiappori A, Williams C, Simon G, Antonia S, Gray J, Litschauer S, Tetteh L, Neuger A, Song L, Rawal B, Schell MJ, Bepler G (2010). Phase I/II study of the Src inhibitor dasatinib in combination with erlotinib in advanced non-small-cell lung cancer. J Clin Oncol.

[13] Isakoff SJ, Wang D, Campone M, Calles A, Leip E, Turnbull K, Bardy-Bouxin N, Duvillie L, Calvo E (2014). Bosutinib plus capecitabine for selected advanced solid tumours: results of a phase 1 dose-escalation study. Br J Cancer.

[14] Johnson FM, Bekele BN, Feng L, Wistuba I, Tang XM, Tran HT, Erasmus JJ, Hwang LL, Takebe N, Blumenschein GR, Lippman SM, Stewart DJ (2010). Phase II study of dasatinib in patients with advanced non-small-cell lung cancer. J Clin Oncol.

[15] Johnson ML, Riely GJ, Rizvi NA, Azzoli CG, Kris MG, Sima CS, Ginsberg MS, Pao W, Miller VA (2011). Phase II trial of dasatinib for patients with acquired resistance to treatment with the epidermal growth factor receptor tyrosine kinase inhibitors erlotinib or gefitinib. J Thorac Oncol.

[16] Kluger HM, Dudek AZ, McCann C, Ritacco J, Southard N, Jilaveanu LB, Molinaro A, Sznol M (2011). A phase 2 trial of dasatinib in advanced melanoma. Cancer.

[17] Mayer EL, Baurain JF, Sparano J, Strauss L, Campone M, Fumoleau P, Rugo H, Awada A, Sy O, Llombart-Cussac A (2011). A phase 2 trial of dasatinib in patients with advanced HER2-positive and/or hormone receptor-positive breast cancer. Clin Cancer Res.

[18] McNeish IA, Ledermann JA, Webber L, James L, Kaye SB, Hall M, Hall G, Clamp A, Earl H, Banerjee S, Kristeleit R, Raja F, Feeney A, Lawrence C, Dawson-Athey L, Persic M (2014). A randomised, placebo-controlled trial of weekly paclitaxel and saracatinib (AZD0530) in platinum-resistant ovarian, fallopian tube or primary peritoneal cancerdagger. Ann Oncol.

[19] Miller AA, Pang H, Hodgson L, Ramnath N, Otterson GA, Kelley MJ, Kratzke RA, Vokes EE, Cancer, Leukemia Group B (2010). A phase II study of dasatinib in patients with chemosensitive relapsed small cell lung cancer (Cancer and Leukemia Group B 30602). J Thorac Oncol.

[20] Reardon DA, Vredenburgh JJ, Desjardins A, Peters KB, Sathornsumetee S, Threatt S, Sampson JH, Herndon JE, Coan A, McSherry F, Rich JN, McLendon RE, Zhang S, Friedman HS (2012). Phase 1 trial of dasatinib plus erlotinib in adults with recurrent malignant glioma. J Neurooncol.

[21] Reddy SM, Kopetz S, Morris J, Parikh N, Qiao W, Overman MJ, Fogelman D, Shureiqi I, Jacobs C, Malik Z, Jimenez CA, Wolff RA, Abbruzzese JL, Gallick G, Eng C (2015). Phase II study of saracatinib (AZD0530) in patients with previously treated metastatic colorectal cancer. Invest New Drugs.

[22] Schilder RJ, Brady WE, Lankes HA, Fiorica JV, Shahin MS, Zhou XC, Mannel RS, Pathak HB, Hu W, Alpaugh RK, Sood AK, Godwin AK (2012). Phase II evaluation of dasatinib in the treatment of recurrent or persistent epithelial ovarian or primary peritoneal carcinoma: a Gynecologic Oncology Group study. Gynecol Oncol.

[23] Sharma MR, Wroblewski K, Polite BN, Knost JA, Wallace JA, Modi S, Sleckman BG, Taber D, Vokes EE, Stadler WM, Kindler HL (2012). Dasatinib in previously treated metastatic colorectal cancer: a phase II trial of the University of Chicago Phase II Consortium. Invest New Drugs.

[24] Jonasch E, Gao J, Rathmell WK (2014). Renal cell carcinoma. BMJ.

[25] Motzer RJ, Jonasch E, Agarwal N, Beard C, Bhayani S, Bolger GB, Chang SS, Choueiri TK, Costello BA, Derweesh IH, Gupta S, Hancock SL, Kim JJ, Kuzel TM, Lam ET, Lau C (2015). Kidney cancer, version 3. 2015. J Natl Compr Canc Netw.

[26] Rini BI, Atkins MB (2009). Resistance to targeted therapy in renal-cell carcinoma. Lancet Oncol.

[27] Garnett MJ, Edelman EJ, Heidorn SJ, Greenman CD, Dastur A, Lau KW, Greninger P, Thompson IR, Luo X, Soares J, Liu Q, Iorio F, Surdez D, Chen L, Milano RJ, Bignell GR (2012). Systematic identification of genomic markers of drug sensitivity in cancer cells. Nature.

[28] Suwaki N, Vanhecke E, Atkins KM, Graf M, Swabey K, Huang P, Schraml P, Moch H, Cassidy AM, Brewer D, Al-Lazikani B, Workman P, De-Bono J, Kaye SB, Larkin J, Gore ME (2011). A HIF-regulated VHL-PTP1B-Src signaling axis identifies a therapeutic target in renal cell carcinoma. Sci Transl Med.

[29] Araujo J, Logothetis C (2010). Dasatinib: a potent SRC inhibitor in clinical development for the treatment of solid tumors. Cancer Treat Rev.

[30] Buettner R, Mesa T, Vultur A, Lee F, Jove R (2008). Inhibition of Src family kinases with dasatinib blocks migration and invasion of human melanoma cells. Mol Cancer Res.

[31] Homsi J, Cubitt CL, Zhang S, Munster PN, Yu H, Sullivan DM, Jove R, Messina JL, Daud AI (2009). Src activation in melanoma and Src inhibitors as therapeutic agents in melanoma. Melanoma Res.

[32] Nam S, Kim D, Cheng JQ, Zhang S, Lee JH, Buettner R, Mirosevich J, Lee FY, Jove R (2005). Action of the Src family kinase inhibitor, dasatinib (BMS-354825), on human prostate cancer cells. Cancer Res.

[33] Shor AC, Keschman EA, Lee FY, Muro-Cacho C, Letson GD, Trent JC, Pledger WJ, Jove R (2007). Dasatinib inhibits migration and invasion in diverse human sarcoma cell lines and induces apoptosis in bone sarcoma cells dependent on SRC kinase for survival. Cancer Res.

[34] Byers LA, Sen B, Saigal B, Diao L, Wang J, Nanjundan M, Cascone T, Mills GB, Heymach JV, Johnson FM (2009). Reciprocal regulation of c-Src and STAT3 in non-small cell lung cancer. Clin Cancer Res.

[35] Shah NP, Kasap C, Weier C, Balbas M, Nicoll JM, Bleickardt E, Nicaise C, Sawyers CL (2008). Transient potent BCR-ABL inhibition is sufficient to commit chronic myeloid leukemia cells irreversibly to apoptosis. Cancer Cell.

[36] Johnston PA, Grandis JR (2011). STAT3 signaling: anticancer strategies and challenges. Mol Interv.

[37] Sen B, Saigal B, Parikh N, Gallick G, Johnson FM (2009). Sustained Src inhibition results in signal transducer and activator of transcription 3 (STAT3) activation and cancer cell survival via altered Janus-activated kinase-STAT3 binding. Cancer Res.

[38] Yu H, Jove R (2004). The STATs of cancer-new molecular targets come of age. Nat Rev Cancer.

[39] Yue P, Turkson J (2009). Targeting STAT3 in cancer: how successful are we?. Expert Opin Investig Drugs.

[40] Tyner JW, Bumm TG, Deininger J, Wood L, Aichberger KJ, Loriaux MM, Druker BJ, Burns CJ, Fantino E, Deininger MW (2010). CYT387, a novel JAK2 inhibitor, induces hematologic responses and normalizes inflammatory cytokines in murine myeloproliferative neoplasms. Blood.

[41] Lehar J, Stockwell BR, Giaever G, Nislow C (2008). Combination chemical genetics. Nat Chem Biol.

[42] Johnson R, Halder G (2014). The two faces of Hippo: targeting the Hippo pathway for regenerative medicine and cancer treatment. Nat Rev Drug Discov.

[43] Lu L, Li Y, Kim SM, Bossuyt W, Liu P, Qiu Q, Wang Y, Halder G, Finegold MJ, Lee JS, Johnson RL (2010). Hippo signaling is a potent *in vivo* growth and tumor suppressor pathway in the mammalian liver. Proc Natl Acad Sci U S A.

[44] Zhou D, Conrad C, Xia F, Park JS, Payer B, Yin Y, Lauwers GY, Thasler W, Lee JT, Avruch J, Bardeesy N (2009). Mst1 and Mst2 maintain hepatocyte quiescence and suppress hepatocellular carcinoma development through inactivation of the Yap1 oncogene. Cancer Cell.

[45] Mizuno T, Murakami H, Fujii M, Ishiguro F, Tanaka I, Kondo Y, Akatsuka S, Toyokuni S, Yokoi K, Osada H, Sekido Y (2012). YAP induces malignant mesothelioma cell proliferation by upregulating transcription of cell cycle-promoting genes. Oncogene.

[46] Cancer Genome Atlas Research N (2013). Comprehensive molecular characterization of clear cell renal cell carcinoma. Nature.

[47] Subramanian A, Tamayo P, Mootha VK, Mukherjee S, Ebert BL, Gillette MA, Paulovich A, Pomeroy SL, Golub TR, Lander ES, Mesirov JP (2005). Gene set enrichment analysis: a knowledge-based approach for interpreting genome-wide expression profiles. Proc Natl Acad Sci U S A.

[48] Shao DD, Xue W, Krall EB, Bhutkar A, Piccioni F, Wang X, Schinzel AC, Sood S, Rosenbluh J, Kim JW, Zwang Y, Roberts TM, Root DE, Jacks T, Hahn WC (2014). KRAS and YAP1 converge to regulate EMT and tumor survival. Cell.

[49] Kapoor A, Yao W, Ying H, Hua S, Liewen A, Wang Q, Zhong Y, Wu CJ, Sadanandam A, Hu B, Chang Q, Chu GC, Al-Khalil R, Jiang S, Xia H, Fletcher-Sananikone E (2014). Yap1 activation enables bypass of oncogenic Kras addiction in pancreatic cancer. Cell.

[50] Horiguchi A, Oya M, Marumo K, Murai M (2002). STAT3, but not ERKs, mediates the IL-6-induced proliferation of renal cancer cells, ACHN and 769P. Kidney Int.

[51] Horiguchi A, Oya M, Shimada T, Uchida A, Marumo K, Murai M (2002). Activation of signal transducer and activator of transcription 3 in renal cell carcinoma: a study of incidence and its association with pathological features and clinical outcome. J Urol.

[52] Jung JE, Lee HG, Cho IH, Chung DH, Yoon SH, Yang YM, Lee JW, Choi S, Park JW, Ye SK, Chung MH (2005). STAT3 is a potential modulator of HIF-1-mediated VEGF expression in human renal carcinoma cells. FASEB J.

[53] Pawlus MR, Wang L, Hu CJ (2014). STAT3 and HIF1alpha cooperatively activate HIF1 target genes in MDA-MB-231 and RCC4 cells. Oncogene.

[54] Sen M, Joyce S, Panahandeh M, Li C, Thomas SM, Maxwell J, Wang L, Gooding WE, Johnson DE, Grandis JR (2012). Targeting Stat3 abrogates EGFR inhibitor resistance in cancer. Clin Cancer Res.

[55] Sen M, Pollock NI, Black J, DeGrave KA, Wheeler S, Freilino ML, Joyce S, Lui VW, Zeng Y, Chiosea SI, Grandis JR (2015). JAK kinase inhibition abrogates STAT3 activation and head and neck squamous cell carcinoma tumor growth. Neoplasia.

[56] Nagaraj NS, Washington MK, Merchant NB (2011). Combined blockade of Src kinase and epidermal growth factor receptor with gemcitabine overcomes STAT3-mediated resistance of inhibition of pancreatic tumor growth. Clin Cancer Res.

[57] Sen M, Thomas SM, Kim S, Yeh JI, Ferris RL, Johnson JT, Duvvuri U, Lee J, Sahu N, Joyce S, Freilino ML, Shi H, Li C, Ly D, Rapireddy S, Etter JP (2012). First-in-human trial of a STAT3 decoy oligonucleotide in head and neck tumors: implications for cancer therapy. Cancer Discov.

[58] Rosenbluh J, Nijhawan D, Cox AG, Li X, Neal JT, Schafer EJ, Zack TI, Wang X, Tsherniak A, Schinzel AC, Shao DD, Schumacher SE, Weir BA, Vazquez F, Cowley GS, Root DE (2012). beta-Catenin-driven cancers require a YAP1 transcriptional complex for survival and tumorigenesis. Cell.

[59] Carey MS, Agarwal R, Gilks B, Swenerton K, Kalloger S, Santos J, Ju Z, Lu Y, Zhang F, Coombes KR, Miller D, Huntsman D, Mills GB, Hennessy BT (2010). Functional proteomic analysis of advanced serous ovarian cancer using reverse phase protein array: TGF-beta pathway signaling indicates response to primary chemotherapy. Clin Cancer Res.

[60] Liao Y, Smyth GK, Shi W (2013). The Subread aligner: fast, accurate and scalable read mapping by seed-and-vote. Nucleic Acids Res.

[61] Liao Y, Smyth GK, Shi W (2014). featureCounts: an efficient general purpose program for assigning sequence reads to genomic features. Bioinformatics.

