# Supplementary material for: Src and STAT3 inhibitors synergize to promote tumor inhibition in renal cell carcinoma
Source: Oncotarget. 2015 Nov 26;6(42):44675–87. doi: 10.18632/oncotarget.5971 (PMC4792584; doi:10.18632/oncotarget.5971)
Supplement: Supplementary file 1 [file oncotarget-06-44675-s001.pdf]

SUPPLEMENTARY FIGURES AND TABLE

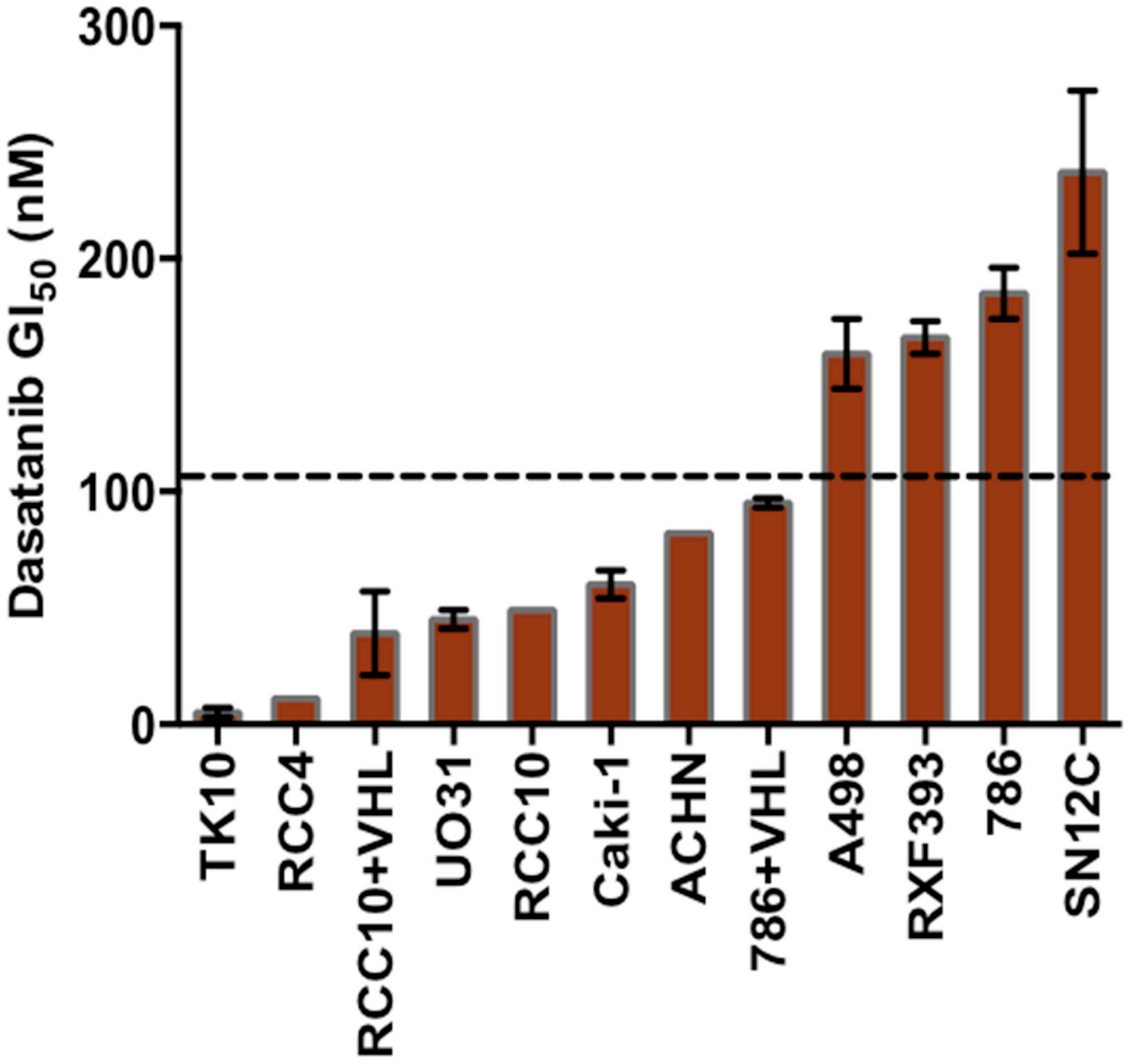

Supplementary Figure 1 (related to Figure 1): Sensitivity of RCC cells to dasatinib. RCC cell lines were treated for 5 days with varying concentrations of dasatinib to determine GI50. Dashed line indicates physiologically relevant dose of dasatinib in patients.

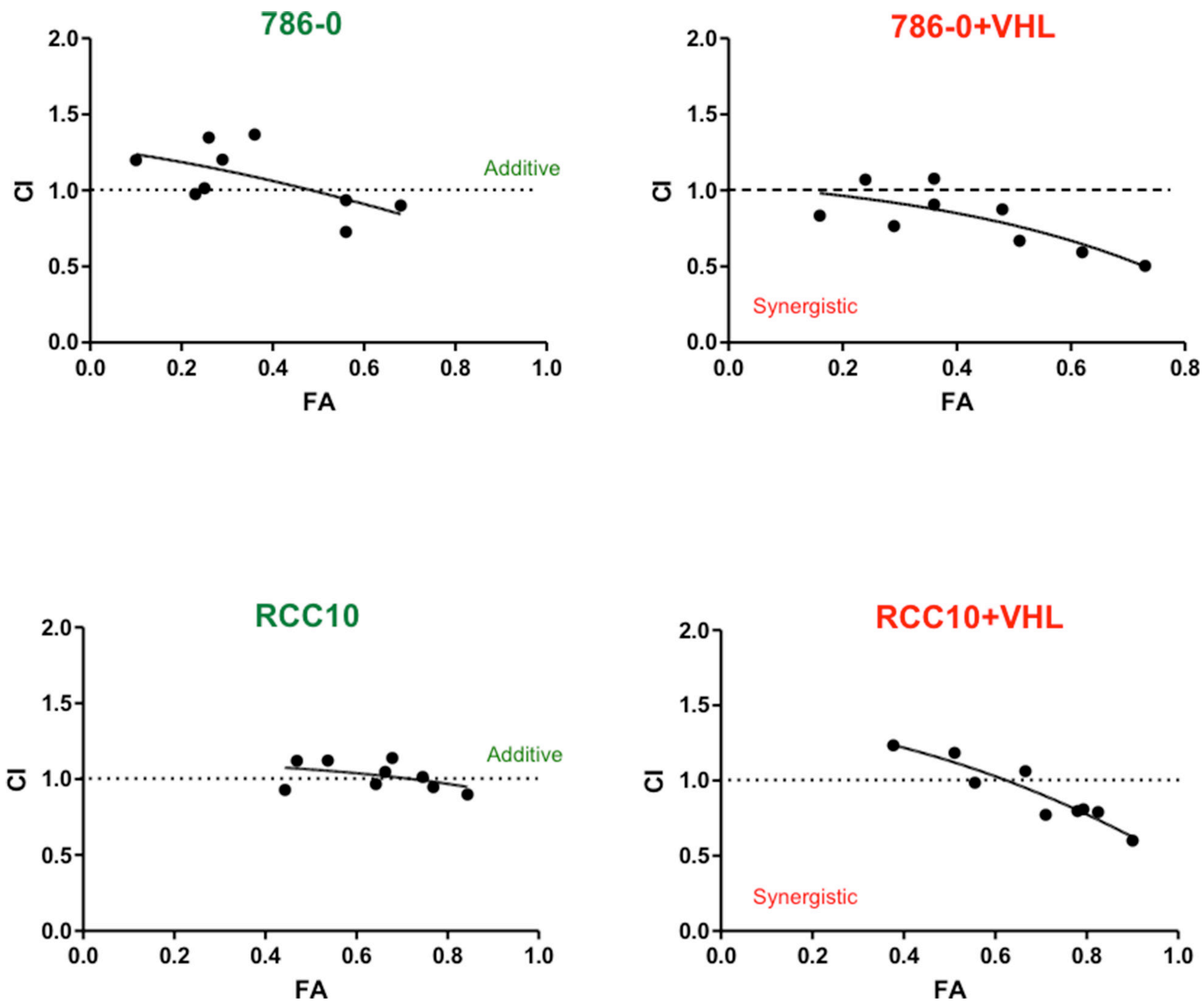

Supplementary Figure 2 (related to Figure 2): Therapeutic activity of combined inhibition of Src an STAT3 in RCC cells. Growth of the indicated RCC cells were analyzed after 5 days of treatment with dasatinib and CYT387. Combination index (CI) were determined by using the Chou-Talalay method (CompuSyn software) for drug combinations with a fractional effect (FA) between 0.2 and 0.9 (20-90% of cell growth inhibition relative to control). CI values <1 indicates drug synergy.

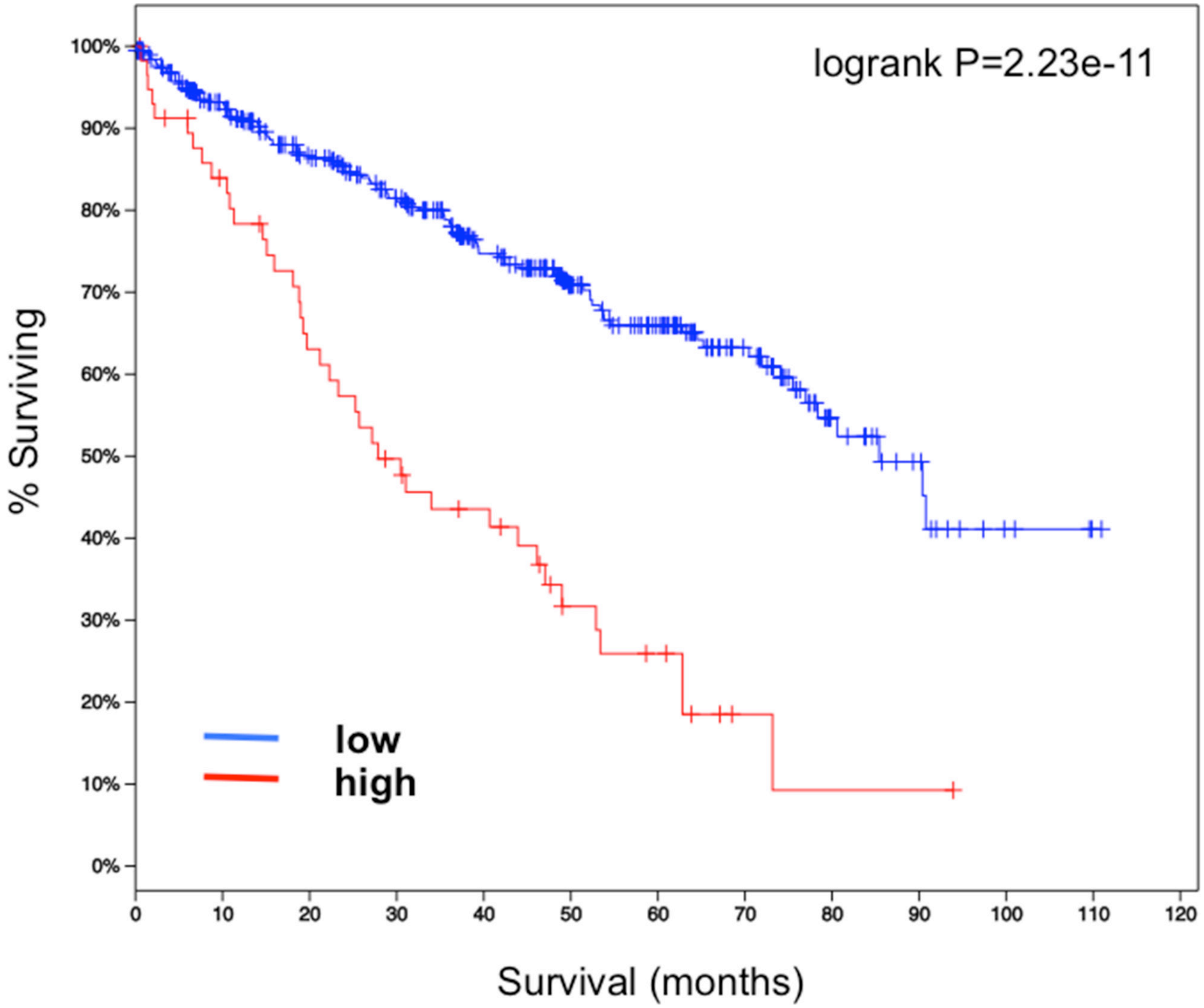

Supplementary Figure 3 (related to Figure 4): Target genes of YAP1 and TAZ are associated with poor survival in RCC patients. Kaplan-Meier survival plots as a function of relative expression of CDK1, AURKA, AURKB, CCNB1 and CCNB2 mRNA in the TCGA kidney cancer cohort of 499 patient tumors (p=2.23e-11).

Supplementary Table S1: Gene Set Enrichment Analysis (GSEA) of genes correlating with sensitivity to combination treatment with dasatinib and CYT387 (related to Figure 4). Table of gene sets from MSigDB (C2) collection enriched among genes downregulated by the dasatinib-CYT387 combination treatment in ACHN cells with normalized enrichment score (NES) ranked by *p*-value

See supplementry file 1.
